# IMMUNIZATION AND VACCINE DEVELOPMENT: Progress towards High and Equitable Immunization Coverage in the Africa Region

**Published:** 2018-07-02

**Authors:** Richard Mihigo, Joseph Okeibunor, Balcha Masresha, Pascal Mkanda, Alain Poy, Felicitas Zawaira, Joseph Cabore

**Affiliations:** WHO Regional Office for Africa

**Keywords:** Immunization, Vaccines, Equity, Coverage, African Region

## Abstract

**Objective:**

This paper gives a brief update on the status of the immunization and vaccine development in the WHO African Region. It also highlights the progress on the control, elimination or eradication of vaccine preventable diseases in the African Region.

**Method:**

The paper reviews national immunization programme data as well as WHO-UNICEF Estimates for Immunization Coverage (WUENIC) in the African Region from 2012-2016.

**Results:**

It revealed that there has been considerable success with the development and introduction of new vaccines in the Region. However, uptake of these vaccines has not matched the level of success in new vaccine introduction. This has made the goal of reaching high and equitable immunization coverage a mirage in the Region. Multiple barriers have been blamed for this, chief among which are inadequate commitment of national governments and weak community engagement to immunization service delivery in the Region. Steps are taken to address these issues, including sensitization of government of the African Region to prioritize *Universal Access to Immunization as a Cornerstone for Health and Development in Africa.* This is because it is argued that development efforts are link to the human beings for whom progress is targeted and/or agents that bring about development.

**Conclusion:**

Saving human lives therefore is critical to the realization of development goals. It is important that immunization coverage is universal to achieve the control/elimination of vaccine preventable diseases.

## Introduction

Beyond the known health benefits^1^,^2^, immunization has been associated with a number of other social and development benefits to the individual, household and community. Some of the often-mentioned social benefits of immunization is its contributions to children’s school attendance and educational performance, increases in household incomes and, ultimately, greater national economic growth^3^. It is now well documented that an investment in vaccines and immunization will yield 16 times returns for every one US Dollar cost, taking into account treatment costs and productivity losses^3^. When considering broader economic and social benefits the return on investment for immunization was 44 times the vaccination costs^4^.

In 2012, the World Health Assembly endorsed Global Vaccine Action Plan (GVAP) 2011-2020, as a framework towards optimizing the benefits of immunization and achieving a vision of expanded access to vaccines and immunization in an equitable manner^5^. In 2014, the WHO Regional Committee in its 64^th^ session endorsed the Regional Strategic Plan for Immunization (RSPI) 2014-2020, with similar goals and targets as the GVAP, which includes 90% coverage with three doses of DPT containing vaccine region-wide and in every country, and 80% or more in every district or equivalent administrative unit^5^,^6^. The RSPI has since been used as a guide by countries in developing their Country Comprehensive Multi-Year Plans (CMYP), with a view to strengthening national immunization systems to achieve high and equitable immunization coverage in Africa.

Improving coverage and equity in immunization programmes is a critical element of ensuring immunization for all, in line with the global and regional commitments made under the GVAP and RSPI. Progress has been made in the African Region, with routine immunization coverage for the third dose of Diphtheria-Tetanus-Pertussis (DTP) containing vaccine, a proxy for immunization performance, raised from 57% in 2000 to 74% in 2016^7^. However, in 2016, the number of infants who did not receive the third dose of DTP vaccines in the WHO African Region was estimated to be 94 million out of an annual birth cohort of 34.6 million; approximately 26%. Two third of these children are living in seven countries only: Angola, DR Congo, Ethiopia, Nigeria, South Africa, South Sudan and Uganda. Coverage has stagnated at around 70% for a prolonged period^8^–^11^.

A number of steps have been taken, in the recent past, to move the Region toward achieving its goal of high and equitable immunization coverage. Some among the steps include the facilitation of countries to establish the National Immunization Technical Advisory Groups (NITAG), in order to provide guidance to policy makers and to make evidence-based immunization related policy decisions in the context of local epidemiology.

In February 2016, the WHO African and East Mediterranean Regional Offices, in conjunction with the African Union Commission held the first ever Ministerial Conference on Immunization in Africa (MCIA), with the theme “towards universal immunization coverage as cornerstone for health and development in Africa”. The MCIA aimed at sensitizing the political leaders on the benefits of immunization and their role in achieving the global and regional targets. The conference concluded with delegates endorsing the declaration on “*Universal Access to Immunization as a Cornerstone for Health and Development in Africa*” signalling fierce determination among African leaders to secure the health and prosperity of their societies through immunization^1^.

The Region has also intensified collaboration with UNICEF and other partners at promoting community ownership of national immunization programmes to create sustainable demand for vaccination services. This is particularly important for increasing demand for and uptake of available services through social and behavioral change interventions; ensuring government transparency and accountability; supporting resource mobilization. Other benefits of involving civil societies and community include influencing national health policies and supporting the monitoring and evaluation of effective programmes. Effective engagement of communities is thus essential to ensuring continued progress toward universal access to immunization.

This paper gives a brief update on the progress of the immunization and vaccine development activities towards high and equitable immunization coverage in the African Region. It also highlights the progress on the control, elimination or eradication of vaccine preventable diseases in the WHO African Region.

## Methods

The methodology used for the review of documents was mainly through the documentary approach to generate data to achieve the objective of assessing the progress made in immunization programmes in the African Region. The WHO-UNICEF Estimate for Immunization Coverage (WUENIC), which is published yearly to show trends in coverage on all vaccine antigens was used as the major source of information for this paper. This was supported with programme reports, which documents the challenges prospects in the immunization programmes. These data sources provided the contextual factors of the programme performance with respect to coverage as well as actions taken to improve challenging situations. Publications on immunization in the African Region were also reviewed.

Descriptive statistics were employed in presenting the data. Tables and figures were also used to illustrate some of the findings in the review.

## Progress on Immunization Coverage and Equity

**Figure 1:**
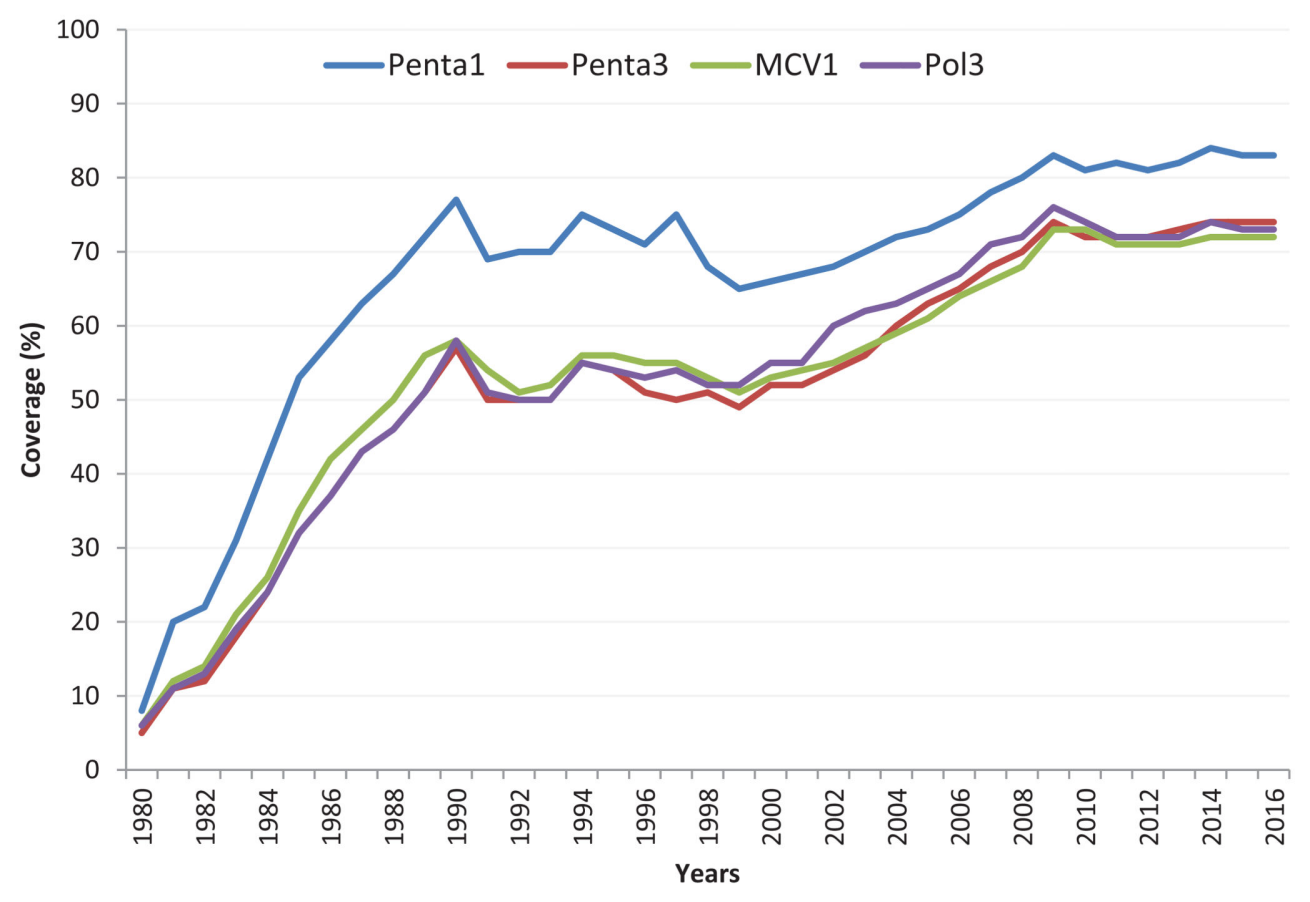
Routine immunization coverage of selected vaccines in the African Region, WUENIC 1980-2015

The 2016 WHO/UNICEF estimate for immunization coverage^7^ revealed a general increase in coverage with all the vaccines (Figure 1). The period 2012 to 2016 showed remarkable improvement (Table 1). Antigens like MCV2 rose from 6% in 2012 to 24% in 2016. Similarly, RCV1 with zero coverage in 2012 had 13% coverage in 2016. The highest proportion of unvaccinated children with Penta3, in 2016, was found in Nigeria (38%). While there is a slight decrease in the number of unvaccinated children in several countries like Guinea, Madagascar, Mali, Mozambique and Niger between 2015 and 2016, the number is however on the rise Angola, Chad, DR Congo, Ethiopia, Nigeria, South Africa, South Sudan and Uganda, known for their high proportion of unimmunized children in the Region. Living in remote rural and urban poor communities were the commonest inequity determinants in immunization. This is followed with mother’s education and wealth status^12^.

As per the country reports to WHO and UNICEF, in 2016, only 8 countries funded 100% of their vaccine costs while 3 countries covered routine immunization costs up to 100%. One country was reported to have not funded routine immunization cost at all.

## Status of Polio Eradication

Overall, there has been significant decrease in number of polio cases and environmental samples; and improved access and supplementary immunization activities (SIA) quality. The last reported case of WPV1 in the Region was in Nigeria with date of onset on 24 July 2014. WPV2 was globally eradicated in 1999 and WPV3 was last reported in Nigeria with date of onset on 10 November 2012. Between July 2014 and July 2016 there was no reported WPV in the Region. However, recent improvement in access to previously insecure areas of Borno, Nigeria, has revealed in 2016 four new cases, most of which were never vaccinated hitherto, due to security challenges.

In terms of circulating vaccine derived polio virus type 2 (cVDPV2) outbreaks there are three major sources of concern in the Region, namely Guinea, Nigeria and DRC. Guinea poses medium to high risk of continuation beyond the switch from the trivalent oral polio vaccine (tOPV) to bivalent oral polio vaccine (bOPV) with risk of spread to neighbouring areas while undetected transmission in Nigeria for over one year remains a great concern in the Lake Chad region.

All the 47 member states have effectively switched from tOPV to bOPV and post switch issues are currently being addressed. Some of the issues include global IPV shortage, intradermal fractional IPV dose use in campaign setting as well as risk of using a live vaccine (mOPV2) with a threat of de-novo VDPV2 emergence. There is also the concern with ensuring high quality mOPV2 responses to VDPVs^13^.

As the polio eradication initiative nears completion, transition planning is developed to protect a polio free world and to ensure that the huge polio investment and assets generated in the course of the initiative contribute to future health goals. Countries, where the most investments were made are encouraged to develop transition plans. Some of the milestones have been reached with polio transition planning. These include the finalization of inventory and mapping of all polio assets; finalization of transitional plans and the start of implementation of transitional plans by January 2017. The main issue here is to ensure government ownership and leadership of the process. WHO is currently giving support to countries for the implementation of the developed milestone to ensure greater ownership of country plans.

The remaining challenges include closing surveillance gaps and ensuring quality outbreak responses; stopping cVDPV/VDPV type 2 emergence and addressing the global shortage of IPV. Other remaining challenges include strengthening national polio certification committees and ensuring government ownership and leadership of the polio transitioning process.

## Progress on New Vaccine Introduction

The Region has recorded significant progress in the introduction of new vaccines. The GVAP target at least 90 low- and middle-income countries introducing one or more new or underutilized vaccines^3^. The success recorded on this target is largely driven by African countries in partnership with Gavi, The Vaccine Alliance. Table 2 reveals that many countries in Africa have introduced multiple new vaccines, such as pneumococcal conjugate vaccine and rotavirus vaccine, at the same time. Pentavalent vaccine (DTP-HepB-Hib), for instance, has been introduced into the routine immunization programmes of all the Member states of WHO African Region. This highlights the high priority of vaccination among political leaders in Africa. The ultimate impact of new vaccines in Africa–as measured by lives saved and illnesses averted–is dependent on the number of children immunized. Countries will need to continue improving routine immunization coverage to achieve the full promise of these vaccines^1^.

## Progress in Disease Control Initiatives

Some successes have been recorded in the control of diseases like meningitis, measles, maternal and neonatal tetanus and yellow fever. This is in spite of the various challenges and public health emergencies and frequent disease outbreaks in the African Region.

### Yellow Fever

Thirty three of the 47 countries in the Africa Region are currently at high risk of yellow fever transmission. The burden of yellow fever, as of 2016, is put at between 840,000 to 1.7 million infections with 84,000 to 170,000 cases and 29,000 to 60,000 deaths. The assumption is that 10% of infections will develop severe symptoms while 35% of severe cases will die.

A simple and effective strategy, which entails vaccination with the one shot vaccine, is being put in place to prevent yellow fever epidemics and secure the vaccine supply for yellow fever outbreak control. The objectives are to control outbreaks, build population immunity, secure vaccine supply and monitor risk, quality, effectiveness and impact. The past decade has demonstrated the changing epidemiology of yellow fever, with countries in Central and East Africa experiencing urban outbreaks, high risk of large deadly outbreaks and high risk of national and international spread. In 2016, Angola reported 2954 suspected cases of YF with 328 deaths from confirmed, probable and suspected yellow fever. The Democratic Republic of Congo had 869 confirmed, probable and suspected cases of yellow fever with 65 deaths.

### Epidemic Meningitis in Africa

The meningitis control strategies include surveillance, case management and vaccination, following WHO revised guidelines, preventive and reactive campaign, introduction of meningococcal vaccine conjugate in routine vaccination. Other control strategies include strengthening laboratory; improve data management, training and research among others.

Mass vaccination with MenA vaccine has been conducted in 16 countries since 2010, targeting persons between 1 and 29 yrs of age. This has brought to almost zero the risk of meningitis A (NmA) epidemics in countries where mass campaigns took place onwards. Cases and deaths due to meningitis in African dropped from 88,199 in 2009 to 30,103 following the introduction of MenAfriVac in 2010. The decline has remained consistent with only 14,338 cases in 2016. Meningitis outbreaks between 2009 and 2016 affected mostly Niger, Nigeria and Burkina Faso. There were also significant decreases of NmA after the introduction of MenAfriVac in 2010. Only one case of NmA was reported in BFA in a vaccinated child aged 8 years old. Over 46 NmA cases were confirmed in 11 countries that conducted MenAfriVac campaigns.

### Measles Elimination

The Region has achieved a significant increase in the first dose of the measles containing vaccine (MCV1) coverage based on WHO-UNICEF Estimates on National Immunization Coverage (WUENIC) between 2001 (54%) and 2009 (73%). However, Regional MCV1 coverage levels have stagnated around 71 – 72% between the years 2009 – 2016.

The regional coverage with the second dose of measles containing vaccine (MCV2) stands at 24% in 2016. As of August 2016, 24 countries have introduced MCV2 in routine EPI. Post- MCV2 introduction evaluation in many countries has shown challenges related to demand creation for MCV2 in the second year of life, weak vaccine management and logistics systems, as well as gaps in the coverage monitoring systems, among others.

As of Dec 2016, 9 countries have introduced rubella vaccine in their national immunisation schedules. In addition, in 2016, eight more countries have conducted wide age range MR supplementary immunization activities (SIAs), which will be followed by introduction into the routine programme. Between January 2015 and Dec 2016, a total of 170.1 million children received measles vaccine through SIAs, in 27 countries.

As of end 2016, 44 of the 47 countries are implementing case-based surveillance for measles. In 2016, a total of 28,823 measles cases were confirmed from across the Region. The Regional incidence of measles was 29.1 cases per million population, with 11 countries having incidence of less than 1 per million. In 2016, twenty countries in the surveillance network attained the targets for the two main surveillance performance indicators (Table 3). The two main surveillance performance indicators are; Non-measles febrile rash illness rate (target of at least 2 per 100,000 population) and the proportion of districts that have investigated at least one suspected case of measles with blood specimen per year (target of 80% or more per year). The non-measles febrile rash illness rate was 2.5 per 100,000 for the Region and 82% of the districts investigated measles cases.

The rate of reporting of non-measles febrile rash illness is 2.5 per 100,000 for the Region (target >/= 2.0 per 100,000), and 82% of the districts in these countries investigated at least one suspected measles case with a blood specimen. Twenty countries met the targets for the two principal surveillance performance monitoring indicators during this period. These included Proportion of reported suspected measles cases from whom blood specimen have been collected ≥80% and proportion of districts that have reported at least 1 suspected case of measles with a blood specimen per year ≥80%)

With the implementation of the key strategies, in the African Region, there has been 78% decline in measles cases since 2000, from a reported number of 520,102 in the year 2000 to 113,938 in 2015. Between 2000 and 2015, the estimated reduction in measles deaths has been 85% for the African region, from an estimated 414,500 deaths in 2000 to an estimated 61,600 deaths in 2015^14^.

### Maternal & Neonatal Tetanus

Maternal and neonatal tetanus constitute yet another set of public health challenge in the Region. Elimination of tetanus is defined as <1 case per 1000 live-births. The recommended strategies for achieving this goal of maternal and neonatal tetanus (MNT) include promotion of clean delivery practices, immunization of women against tetanus targeting pregnant and those in child bearing age group (15-44 years) with a tetanus toxoid-containing vaccine in routine immunization, or provision of at least 3 doses of tetanus toxoid (TT) vaccine through supplemental immunization activities’ (SIAs) targeting women of reproductive age that reside in areas classified as being at high risk for MNT, and case-based surveillance to identify NT cases and deaths as well as the assessment of the risk-status of the area^15^.

As of 2016, 37 of the 47 Member States have been validated for MNT elimination. The remaining countries (Angola, Central African Republic, Chad, Democratic Republic of the Congo, Equatorial Guinea, Guinea, Kenya, Mali, Niger, Nigeria and South Sudan, and the Somali region of Ethiopia) are making efforts to complete needed supplemental immunisation activities in high risk districts. Six out of the ten remaining countries are ready to undergo the validation exercise.

Progress towards achieving the goal has, however, been delayed in the African Region, due in part to slow implementation of the recommended strategies. It is estimated that the current reporting system captures less than 10% of cases. This is because the health care seeking behaviour of most of the affected rural communities are difficult to access, populations rely more on traditional and spiritual healers for such diseases of sudden onset, and health facilities are only visited as the last resort, coupled with the fact that the existing surveillance system is focused more on review of medical records at the health facility level with limited community surveillance component.

## Vaccine Research and Development

Research has also been identified and prioritized for its catalytic effect on reaching development goals. Some steps have been taken to advance the course of research and development. A Strategic Framework for Research in Immunization and Implementation Research is put in place to guide research operations of the immunization programmes of Member States. The Region has been supporting implementation researches to strengthen immunization programmes and move them towards attaining the global and regional immunization goals and targets.

In terms of vaccine development, the WHO Product Development for Vaccines Advisory Committee (PDVAC) is working to accelerate vaccine candidates in Phase 2 of clinical evaluation or earlier, for diseases with substantial burden in low and medium income countries (LMICs), but where no vaccines currently exist, through WHO guidance. Specifically, the objectives are to develop the preferred product characteristics (PPCs); WHO preferences for vaccines to be used in LMICs (indications, target groups and desirable clinical data on safety and efficacy); provide early guidance to developers, 5–10 years from vaccine approval; and ensure that, once licensed, data are available for decision-making on use in populations that need it most. Some typical cases where the WHO has pursued these goals are in the development of the first malaria and Ebola vaccines in the Region.

## Conclusion

Despite the current inability to reach both global and regional immunisation goals, national immunization programmes in the African Region have made significant strides in protecting the populations against vaccine preventable diseases. New and effective vaccines have been successfully introduced in national immunization programmes. Polio eradication has also been on track, except for the recent detection of WPVs in the previously inaccessible and security compromised zone of Northeast Nigeria, which has also been promptly and effectively responded to. Coverage with other vaccines has also been on a steady increase, albeit, stagnating around 70% for a prolonged period, and failing to reach the desired 90% national coverage and 80% in all the districts, due to multiple logistic, systems and behavioural challenges. Some of the challenges are also linked to weak country ownership, inadequate government support for immunization programmes and community demand for immunization services.

The IVD programme has taken various steps, most of which are yielding positive returns, to push national immunization programmes towards achieving the global and regional targets. Countries are being facilitated to establish NITAG to guide the national immunization programmes. Governments were recently sensitized, through the first ever Ministerial Conference on Immunization in Africa (MCIA), on the situation of immunization programmes in the African continent and their role in moving immunization programmes toward attaining high coverage with equity in access to immunization services as a critical pillar for socioeconomic development of the Region.

On the other hand, communities and individuals are increasingly being sensitized to the benefits of immunization and their right to demand immunization services. Together with UNICEF and other partners, in the Region, WHO is regularly working on boosting demand for immunization services. The planning of CMYPs is increasingly being improved to cover communication issues that drive demand creation. Furthermore, results of various implementation research are being utilized in planning the delivery of immunization services, developing effective communication tools and messages, particularly addressing risk communications resulting from adverse events after immunization.

To enhance the capacity of countries to undertake implementation research in support of the immunization programmes, capacity building workshops were conducted for selected French and English-speaking countries respectively. An implementation research guide has also been produced to help the immunization programme managers on the processes of identifying and initiating scientific studies to address implementation issues. In addition to this, the IVD programmes in the Regional Office is in the process of producing a strategic framework for research in immunization, which will go beyond providing guide to doing research but also provide a platform for prioritizing research questions and research resource allocation.

With these, the IVD programme is cautiously optimistic of attaining the global and regional immunization goals by the year 2020. However, this will be with the continued support of all stakeholders, the countries and their partners.

## Figures and Tables

**Figure 2:**
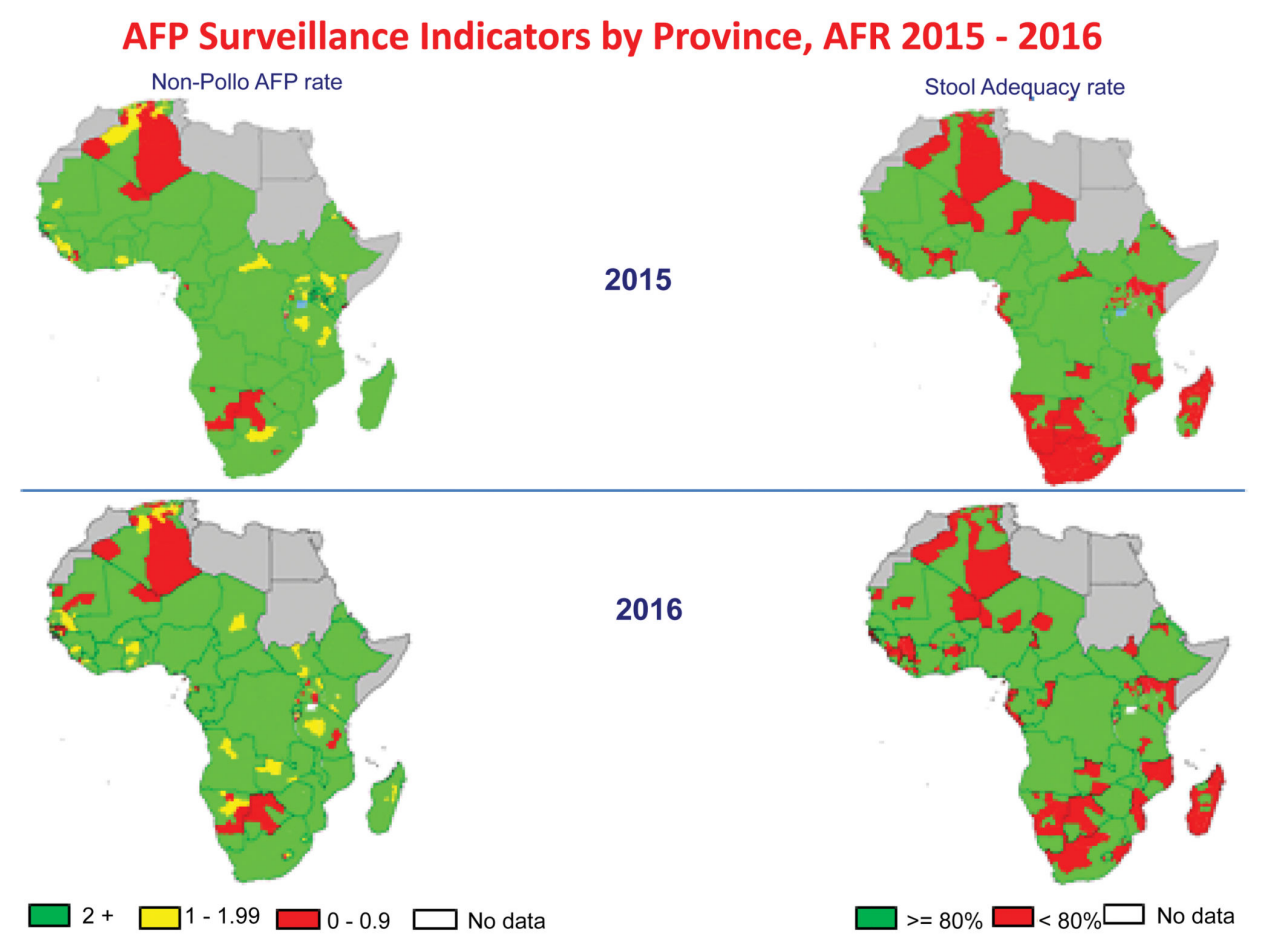
AFP surveillance performance challenges in the Region, as at July 2016

**Table 1:** Routine immunization coverage in African Region, WUENIC, 2016

Vaccine	Coverage by Year				
	2012	201	2014	2015	2016
BCG	80	79	82	80	81
HepB_BD	9	9	9	9	10
Penta1	81	82	84	83	83
Penta2	72	73	74	74	74
MCV1	71	71	72	72	72
MCV2	6	7	11	18	24
YFV	36	40	45	45	45
PCV3	21	35	50	59	65
OPV3	72	72	74	73	73
RCV1	0	4	9	12	13
Rotac	4	12	29	40	45
IPV1				19	41

**Table 2:** Status of introduction of new vaccine in countries of the African Region, 2016

PCV	Rota	HPV In RI	HPV in Demonstration	HepB	Penta	IPV
Angola	Angola	Botswana	Benin	Algeria	Algeria	Algeria
Benin	Botswana	Lesotho	Burkina Faso	Angola	Angola	Benin
Botswana	Burkina Faso	Rwanda	Burundi	Botswana	Benin	Botswana
Burkina Faso	Burundi	Seychelles	Cameroon	Cabo Verde	Botswana	Burundi
Burundi	Cameroon	South Africa	Cote D'Ivoire	Gambia	Burkina Faso	Cameroon
Cameroon	Congo	Uganda	Ethiopia	Mauritania	Burundi	CAR
Congo	Eritrea		Gambia	Namibia	Cabo Verde	Chad
Cote d'Ivoire	Ethiopia		Ghana	Nigeria	Cameroon	Comoros
C AR	Gambia		Kenya	Sao Tome & Principe	C AR	Congo
DRC	Ghana		Liberia	Senegal	Chad	Cote D'Ivoire
Eritrea	Guinea Bissau		Madagascar		Comoros	DRC
Ethiopia	Kenya		Malawi		Congo	Equatorial Guinea
Gambia	Liberia		Mali		Cote D'Ivoire	Ethiopia
Ghana	Madagascar		Mozambique		DRC	Gabon
Guinea Bissau	Malawi		Niger		Equatorial Guinea	Gambia
Kenya	Mali		Senegal		Eritrea	Guinea
Lesotho	Mauritania		Sierra Leaone		Ethiopia	Guinea Bissau
Liberia	Mauritius		Tanzania		Gabon	Kenya
Madagascar	Mozambique		Togo		Ghana	Lesotho
Malawi	Namibia		Zambia		Guinea	Madagascar
Mali	Niger		Zimbabwe		Guinea Bissau	Mauritania
Mauritania	Rwanda				Kenya	Mozambique
Mauritius	Senegal				Lesotho	Namibia
Mozambique	Sierra Leone				Liberia	Niger
Namibia	Sao Tome & Principe				Madagascar	Nigeria
Niger	South Africa				Malawi	Sao Tome & Principe
Nigeria	Swaziland				Mali	Senega;
Rwanda	Tanzania				Mauritania	Seychelles
Sao Tome & Principe	Togo				Mauritius	South Africa
Senegal	Zambia				Mozambique	South Sudan
Sierra Leone	Zimbabwe				Namibia	Swaziland
South Africa					Niger	Uganda
Swaziland					Nigeria	
Tanzania					Rwanda	
Togo					Sao Tome & Principe	
Uganda					Senegal	
Zambia					Seychelles	
Zimbabwe					Sierra Leone	
					South Africa	
					South Sudan	
					Swaziland	
					Tanzania	
					The Gambia	
					Togo	
					Uganda	
					Zambia	
					Zimbabwe	

**Table 3:** Performance of Measles Elimination by Targets

Introduce Rubella Vaccine	Conducted Wide Age MR SIAs	Countries with measles incidence <1/1000000	Countries with surveillance network meeting target
Burkina Faso	Botswana	Botswana	Botswana
Cabo Verde	Cameroon	Cabo Verde	Cameroon
Ghana	Gambia	Comoros	Chad
Mauritius	Kenya	Guinea Bissau	Congo
Rwanda	Namibia	Madagascar	Gabon
Senegal	Sao Tome and Principe	Malawi	Guinea
Seychelles	Swaziland	South Africa	Kenya
Tanzania	Zambia	Swaziland	Lesotho
Zimbabwe		Tanzania	Madagascar
		Zambia	Mali
		Zimbabwe	Mozambique
			Rwanda
			Senegal
			Sierra Leone
			South Africa
			Swaziland
			Togo
			Uganda
			Zimbabwe
